# How French media have portrayed ADHD to the lay public and to social workers

**DOI:** 10.1080/17482631.2017.1298244

**Published:** 2017-05-22

**Authors:** Sébastien Ponnou, François Gonon

**Affiliations:** ^a^ Pôle Limousin d’Action et de Recheche en Intervention Sociale, Limoges, France; ^b^ Laboratoire Education et Diversité en Espaces Francophones, University of Limoges, Limoges, France; ^c^ Institute of Degenerative Disease, CNRS UMR5293, University of Bordeaux, Bordeaux, France

**Keywords:** Attention deficit hyperactivity disorder, newspaper, television programmes, biomedical model, psychodynamic model, content analysis, methylphenidate, over-prescription, medicalization

## Abstract

Two models of attention deficit hyperactivity disorder (ADHD) coexist: the biomedical and the psychosocial. We identified in nine French newspapers 159 articles giving facts and opinions about ADHD from 1995 to 2015. We classified them according to the model they mainly supported and on the basis of what argument. Two thirds (104/159) mainly supported the biomedical model. The others either defended the psychodynamic understanding of ADHD or voiced both models. Neurological dysfunctions and genetic risk factors were mentioned in support of the biomedical model in only 26 and eight articles, respectively. These biological arguments were less frequent in the most recent years. There were fewer articles mentioning medication other than asserting that medication must be combined with psychosocial interventions (14 versus 57 articles). Only 11/159 articles claimed that medication protects from school failure. These results were compared to those of our two previous studies. Thus, both French newspapers and the specialized press read by social workers mainly defended either the psychodynamic understanding of ADHD or a nuanced version of the biomedical model. In contrast, most French TV programmes described ADHD as an inherited neurological disease whose consequences on school failure can be counteracted by a very effective medication.

## Introduction

Mass media have widely discussed attention deficit hyperactivity disorder (ADHD) and have contributed to shape its perception by the public (Bussing et al., 2012). Horton-Salway (2011) examined how UK newspapers have represented ADHD and identified two repertoires: the biological and the psychosocial. The biological repertoire describes ADHD as a neurological pathology and encourages its medication. English-written newspapers reporting scientific studies about ADHD almost exclusively support this repertoire (Gonon, Bézard, Boraud, 2011; Gonon, Konsman, Cohen, & Boraud, 2012). The psychosocial repertoire put forward inadequate parenting practices and social problems such as poor school systems, excessive TV exposure or high level of premature birth. Horton-Solway (2011) observed that the psychosocial repertoire was dominant in UK newspapers during 2000–2009. However, Schmitz, Filippone, and Edelman (2003) identified the biological model of ADHD as dominant in US newspapers during 1988–1997 (Schmitz et al., 2003). Similarly, Swedish newspaper articles published in 1997–1998 about the educational system “could be described as a launch of a campaign in favour of a medical perspective in children’s problems”, including ADHD (Börjesson, 1999, p. 3).

In France, methylphenidate has been approved for ADHD treatment since 1995. This prescription to children was very low until 2002 and then rose sharply at a rate of 11% per year. About 0.5% of French children and adolescents received at least one prescription in 2013. The proponents of this medication have therefore claimed that ADHD is under-diagnosed and under-treated in France since, according to the only study on this topic, ADHD prevalence among 4–17-year-old French children ranges from 3.5 to 5.6% (Lecendreux, Konofal, & Faraone, 2011). However, in France, the biological model of ADHD as a neurological disease has been counteracted by a psychodynamic understanding considering that ADHD symptoms should be seen as a child’s response to emotional distress or as a defence mechanism against depression and trauma (Chagnon, 2010; Delvenne, 2007). From this viewpoint, the medication can be considered as useful if it facilitates a psychotherapeutic approach (Golse & Zigante, 2002).

We have already published two studies investigating how ADHD is portrayed by French media (Bourdaa et al., 2015; Ponnou, Kohout-Diaz, & Gonon, 2015). In the first one, we analysed 60 TV programmes (including news, talk shows and debates, but excluding fiction) broadcast from 1995 to 2010. We tested whether and how they reported on three scientific questions about ADHD: (i) whether ADHD is mainly genetic in origin; (ii) whether methylphenidate treatment decreases the risk of academic underachievement; and (iii) whether brain imaging techniques are able to reveal ADHD in individual patients. Scientific studies demonstrated that genetic factors only mildly contribute to ADHD etiology whereas environmental factors play a central role (Ficks & Waldman, 2009; Sonuga-Barke, 2010). In a recent review of more than 300 molecular genetic studies (Li, Chang, Zhang, Gao, & Wang, 2014, p. 19), the authors concluded: “current findings from genetic studies of ADHD are still inconsistent and inconclusive. One of the reasons that hamper us from obtaining consistent and significant findings is the relatively smaller sample size [of the published studies]”. However, this review also showed that the number of patients included in individual studies was multiplied 10-fold from the early 1990s to the late 2000s (Li et al., 2014). Epidemiological studies have shown that ADHD children are at an elevated risk of academic underachievement and that psychostimulant medication does not decrease this long-term risk (Sharpe, 2014). Despite premature claims (Dougherty et al., 1999), brain-imaging techniques cannot be used to diagnose ADHD. Nevertheless, French TV programmes reporting on these three questions preferentially supported opinions defended by initial studies although they have been contradicted by subsequent ones. Even in the most recent period (2007–2010), nine out of 10 TV programmes still claimed that ADHD is a genetic disease, that medication counteracts the risk of academic underachievement and that brain imaging can be used as a diagnostic tool. Thus, the biological repertoire dominated French TV programmes reporting on ADHD without obvious change for 16 years (Bourdaa et al., 2015).

In the second study, we analysed 93 articles published from 1995 to 2012 in French specialized journals read by social workers and discussing ADHD (Ponnou et al., 2015). Among them, 19 mentioned genetic factors, but only two erroneously claimed that they represent the main cause of ADHD. Only four articles discussed the effect of medication on the risk of academic underachievement and only one claimed that medication is very effective. None suggested that brain imaging might be used to diagnose ADHD. We identified in this specialized press two models of ADHD symptoms: the biomedical and the psychodynamic model (Ponnou et al., 2015). Both discourses did not evolve in parallel: the psychodynamic understanding was dominant in the early 2000s while the biomedical model dominated during the most recent years (2009–2012). Finally, the scientific literature has widely documented the environmental risk factors that strongly contribute to ADHD prevalence: exposure to heavy metals and other chemicals (Froehlich et al., 2011; Needleman et al., 1979), premature birth (Linnet et al., 2006; Szatmari, Saigal, Rosenbaum, Campbell, & King, 1990), maltreatment, parents suffering from mental disorders, poor interactions between parents and children (Biederman, Faraone, & Monuteaux, 2002; Biederman et al., 1995; Galera et al., 2011; Schneider & Eisenberg, 2006; Tallmadge & Barkley, 1983), low economic status of the family, low educational level of the parents, young maternal age at birth (Froehlich et al., 2007; Galera et al., 2011; Schneider & Eisenberg, 2006), and excessive exposure to television and video games (Christakis, Zimmerman, DiGiuseppe, & McCarty, 2004; Swing, Gentile, Anderson, & Walsh, 2010). Unfortunately, these risks are never mentioned in French specialized journals and TV programmes (Ponnou et al., 2015).

In the present study, we analysed the representation of ADHD in the French general press from 1995 to 2015, and compared it with those provided by French TV programmes and the specialized press read by social workers. In line with our previous studies we classified each newspaper article as preferentially defending the biomedical or the psychodynamic model. We also considered a third class of articles citing experts defending both. For each article we also investigated how they reported on questions defined in our previous studies about genetic etiology, academic underachievement, medication and brain imaging.

## Methods

### Data collection

We collected press articles dealing with ADHD in four major French national daily newspapers (*La Croix, Le Figaro, Le Monde* and *Libération*), three weekly newspapers (*L’Express, Le Nouvel Observateur* and *Le Point*) and two major regional dailies (*Le Progrès* and *Sud Ouest*). We selected national journals on the basis of their circulation ranking, after exclusion of specialized journals such as *L’Equipe* (sport), *Les Echos* (economy) or *TV Magazine* (TV programmes). We selected two regional dailies that widely differ regarding their distribution area. We used the EUROPRESSE database (www.europresse.com) to collect all articles published from January 1995 to December 2015 in these nine newspapers and containing the keyword “hyperactivity/hyperactive”. We used this keyword rather than the French translation of ADHD (TDAH) because it is much less common in the French media than “hyperactivity”. We identified 709 articles and, then, excluded: (i) articles where the term “hyperactivity” is used to describe the behaviour of normal people (e.g. hyperactivity of a politician); (ii) articles where hyperactivity is only associated with another disease (e.g. hyperactivity in children with autism); and (iii) articles that marginally mentioned ADHD and provided no information or opinion about it. We kept 159 articles dealing with “hyperactivity” considered as a disorder or, at least, a symptom. The list is available on request to the first author.

### Content analysis

We classified each article into three categories: (i) those that mainly support the biomedical model of ADHD; (ii) those that mainly defend the psychodynamic understanding; and (iii) those that voice both views. We also analysed the discourse about ADHD through five detailed questions that may or may not be discussed in each article. The first one is related to the contribution of genetic factors to ADHD etiology. We classified articles reporting on this question as (i) defending that genetic factors are the main contributors; (ii) defending that environmental rather than genetic factors play a major role; and (iii) giving voice to experts defending both views. The second question is related to the causes of ADHD beside genetic factors. We considered four classes of causes: (i) neurological or neurodevelopmental dysfunctions; (ii) premature birth; (iii) inappropriate education (either provided by parents or school); and (iv) other causes (anaesthesia, poverty, adoption and excessive exposure to TV, pesticides, alcohol or food additives). The third question is related to ADHD treatment: when an article tackles this question does it favour medication, psychotherapy or a combined treatment? The fourth question is related to the effectiveness of medication to decrease the risk of academic underachievement. The fifth question concerns articles discussing the use of brain imaging to diagnose ADHD. We identified in each article whether or not it discussed these five questions. Therefore, for each article we filled a reading table of 23 items. This reading table was first coded by one author (FG) and then independently coded by the other author (SP). The few disagreements were resolved by discussion. The reading table of the 159 articles is given in supplementary material (Ponnou-data.xlsx). All articles were written by journalists. They often gave voice to health professionals (medical doctors, psychologists, psychoanalysts, social workers) and to parents, either as short quotations or full interviews, but not to children. However, we did not quantify these quotations in the present study.

## Results

### Dominant ADHD model in French newspaper articles

Among the 159 articles we found only 55 explicitly defending the psychodynamic model, either alone (26 articles) or voicing both models (29 articles). About two-thirds of articles were classified as mainly supporting the biomedical model. This percentage was remarkably stable over two decades (Table I).

**Table I.** Number of newspaper articles mainly defending either model or both.

Model	1995–2000	2001–2005	2006–2010	2011–2015	Total
Biomedical	10 (66.7%)	32 (69.6%)	28 (62.2%)	34 (64.2%)	104
Psychodynamic	1	6	13	6	26
Both	4	8	4	13	29
Total	15	46	45	53	159

### Contribution of genetic factors to ADHD

Only 25 articles out of 159 discussed the contribution of genetic factors to ADHD etiology (Table II). Among them, eight articles asserted that they play a major role, as illustrated in extract 1.

The contribution of genetic factors to ADHD is likely strong, according to experts. Studies suggest that the heritability of ADHD might range between 60% and 80%. This means that an ADHD patient might have a 60 to 80% chance of inheriting it through genes. (Extract 1: *Le Monde*, 16 November 2009)

**Table II.** ADHD risk factors highlighted in French newspapers.

Causes of ADHD		1995–2000	2001–2005	2006–2010	2011–2015	Total
ADHD is mainly genetic	Yes	0	2	5	1	8
	No	0	3	0	2	5
	Yes + no	0	7	3	2	12
Neurological deficit		3	10	8	5	26
Premature birth		0	3	2	1	6
Inadequate education		3	9	14	9	35
Other causes		0	1	9	16	26

Notice that the inference raised in this quotation is scientifically wrong: a high heritability does not prove a strong genetic causation because heritability studies cannot disentangle pure genetic effects from gene–environment interactions (Freitag, Rohde, Lempp, & Romanos, 2010; Visscher, Hill, & Wray, 2008). Moreover, estimates of ADHD heritability were strongly influenced by assessment instruments and rating scale (Freitag et al., 2010). For example, whereas most twin studies using rating scales reported high heritability estimates (60–80%) (Freitag et al., 2010), objective measurements of inattention and impulsivity led to lower estimates (30–36%) (Freitag et al., 2010; Heiser et al., 2006).

In contrast, five articles, in agreement with the present scientific consensus (Ficks & Waldman, 2009; Li et al., 2014; Sonuga-Barke, 2010), challenged this claim. For example we found in *Le Figaro* (19 February 2004): “It is obvious that hyperactive children suffering from attention deficit are increasingly numerous. One cannot put forward genetic mutations. Therefore, we must wonder about societal changes.” Twelve articles voiced both opinions. Of the eight articles supporting a major genetic causation for ADHD, only one echoed a scientific study. On 2 October 2010, the most respected French newspaper *Le Monde* echoed a study published in the prestigious medical journal *The Lancet* and claiming that DNA deletions or duplications are twice as frequent in ADHD children as in unaffected ones (Williams et al., 2010). Actually, the medical relevance of this study was weak: these DNA alterations were detected in 12.5% of ADHD children and 7.5% of unaffected ones. However, the newspaper article did not give these raw data and did not comment on this intrinsic weakness. Moreover, this difference in DNA alterations was not found by another study published in June 2010 (Elia et al., 2010), but this previous finding was not echoed by *Le Monde*.

### Causes of ADHD given in the French press, other than genetic factors

Among the 159 articles examined, 26 represented ADHD as caused by neurological dysfunctions. For example in the *Sud Ouest* (30 June 2014) ADHD is said to be: “an immature neurological functioning in the frontal area [of the brain], that is supposed to control impulsivity”. Only one article published in 2009 discussed these possible dysfunctions and challenged this conclusion on the basis of scientific arguments: differences observed with brain imaging techniques between ADHD children and controls are mild, often inconsistent between studies and do not prove that they are the cause rather than the consequence of the disorder (*Libération*, 31 August 2009). This representation of ADHD as resulting from neurological dysfunctions reached its maximal frequency during an early period (2001–2005) and then declined in absolute number (Table II) and even more in percentage of articles (from 21.7% in 2001–2005 to 9.4% in 2010–2015). This is surprising at first glance because we observed no parallel time evolution in the percentage of articles classified as mainly defending the biomedical model. It seems that the proponents of this model became more prudent during the recent period and refrained from putting forward weak neurobiological arguments. In line with this trend, the French National Authority for Health (HAS) released on February 2015 a report about ADHD management that also discussed its etiology in light of biological, social, and psychodynamic viewpoints (HAS, 2015). Moreover, the merits and weaknesses of the biological pieces of evidence are fairly discussed and the HAS report concluded that the biological causes of ADHD are still poorly understood. This report was widely discussed in French newspapers. Most articles pointed out that ADHD symptoms reveal a real disorder needing care, but did not put forward biological arguments. Some others even gave voice to a psychoanalyst, who is also a psychiatrist: “ADHD has no scientific validity, it is a social construct” (*Libération*, 12 February 2015).

Although prematurity at birth is a recognized risk factor for subsequent ADHD (Galera et al., 2011; Linnet et al., 2006), it is mentioned by only five newspaper articles (Table II). For example, *La Croix* (25 May 2011) put: “A Swedish study published in the journal *Pediatrics* highlights that premature birth is linked to the risk of receiving an ADHD treatment some years later.” It must be noted, however, that prematurity is not a major concern in France: the rate of premature birth is much lower (5.8%) than in the USA (12.7%) (Goldenberg, Culhane, Iams, & Romero, 2008).

Inadequate parenting and/or inappropriate school systems are the most often mentioned risk factors contributing to ADHD (35 articles, Table II). Two examples are given in the second extract. The first one translates the opinion of a psychoanalyst who is also a psychiatrist, but the second one comes from an article classified as defending the biomedical model.

Since the late 1990s psychiatrists have made up a new disorder to describe children who are disruptive at school: attention deficit hyperactivity disorder. And, then, they have diagnosed children who present symptoms of inattention such as squirming in their seats, moving their feet or challenging all forms of authority. In other words they have transformed into a mental disorder, and then into a world-wide epidemic, what was nothing but a disturbance linked to difficult relationships between children, parents, educators and teachers. (Extract 2a. *Le Monde*, 13 February 2015)

Canadian scientists … have performed a prospective study on 4874 children who lived with both their parents in 1994. They then compared the rate of psychostimulant prescription in 2000 between children still living with their two parents and children whose parents had divorced. The results showed that 6.1% of the children whose parents had divorced were on medication whereas only 3.3% of those living with the parental couple were. (Extract 2b. *Le Figaro*, 5 June 2007)

These opinions are consistent with the scientific literature. Maladaptive parenting and/or parental psychopathology are common among parents of ADHD children (Biederman et al., 2002; Modesto-Lowe, Danforth, & Brooks, 2008). Likewise, the US school system contributes to the rise of the ADHD diagnosis. First, teachers are involved in the detection and diagnosis of ADHD children when completing the Conners Teacher’s Rating Scale (Phillips, 2006). Second, children who are relatively young for the grade (i.e. born just before the cut-off) are at a greater risk of ADHD diagnosis than children who are relatively old for the grade (i.e. born just after the cut-off) (Evans, Morrill, & Parente, 2010). And third, children living in states with more stringent school accountability laws are more likely to be diagnosed with ADHD (Bokhari & Schneider, 2011). Similar studies are lacking in France, but it has been noted that children at risk of ADHD symptoms exhibit the same characteristics as those at risk of school failure at the end of the first year of the primary school: to be a boy rather than a girl, to be born in December rather than in January and to live in a family with a low economic status (FNAME, 2012).

Some newspaper articles put forward other risk factors that may contribute to ADHD. Ten articles mentioned an excessive exposure to television and/or video games and this is consistent with the scientific literature (Landhuis, Poulton, Welch, & Hancox, 2007; Weiss, Baer, Allan, Saran, & Schibuk, 2011; Zimmerman & Christakis, 2007). For example *Le Monde* (3 September 2014) reported: “Several scientifically sound studies have demonstrated that excessive exposure to screens [TV or video games] is linked to attention deficit.” Four articles highlighted the possible risk linked to the excessive consumption of certain food (sugar, colouring, additives), but another article, in agreement with short-term studies (Wolraich, Wilson, & White, 1995), stated that sugar is not a significant risk. Exposure to chemicals (pesticides, anaesthetics) during first years and to alcohol or nicotine during foetal life were mentioned by five articles. Finally, only two articles mentioned a major social risk factor: the low economic status of the family (Froehlich et al., 2007).

### Therapeutic options for ADHD given in the French press

Among the 159 articles we examined, 14 mentioned only medication for the treatment of ADHD (Table III). For example, an article published in *Le Progrès* (26 November 2002) stated about ADHD: “A diagnosis of ADHD justifies a methylphenidate prescription.” Eighteen articles cited various alternative options only (e.g. psychotherapy, neurofeedback, meditation). The vast majority (57 articles, Table III) emphasized that ADHD treatment requires a multidisciplinary approach combining medication with psychosocial treatments and services (e.g. cognitive/behavioural therapy, parental training, family therapy, school services) as stated in extract 3:

Methylphenidate, the only drug authorized [in France] to treat ADHD, has been put at its rightful place: never as first-line therapy, always in combination with therapies and only if the child and his/her parents are in real need of it to silence symptoms until the troubles ease. (Extract 3. *Le Figaro*, 12 February 2015, commenting on an official recommendation about ADHD that had just been released)

**Table III.** Therapeutic options and medication criticisms.

	1995–2000	2001–2005	2006–2010	2011–2015	Total
Medication alone	0	6	7	1	14
Psychotherapy	1	4	8	5	18
Combined	5	17	10	25	57
*Medication criticism*	9 (60%)	9 (19.6%)	10 (22.2%)	16 (30.2%)	44

We also found 44 articles questioning psychostimulant medication (Table 3). In the early period (1995–2000) 60% of the articles expressed the fear that this medication might have unknown long-term side effects and might be diverted and abused. Most articles also emphasized that psychostimulant medications are overprescribed in the USA and quoted experts that warned against over-prescription in France. After this early period, these criticisms were less frequent but did not disappear (Table III). These criticisms were expressed in 24 out of the 104 articles classified as mainly defending the biomedical model (see a typical example in extract 4):

The effects of this drug [methylphenidate] are worrying and the future of medicated children reaching adulthood is unknown. On medication, growth is slowed down, heart rate and blood pressure are increased, and psychotic symptoms may occur. In France and other European countries cases of abuse and addiction are documented. This prescription should be the last resort for treating only the most severe cases. (Extract 4, *Le Point*, 9 May 2013)

The number of articles questioning medication is also given in % of the total number of articles in the same period (see Table I).

### Long-term risks associated with ADHD

Academic underachievement was frequently cited as the inescapable consequence of untreated ADHD (Table IV) and this is consistent with the scientific literature (Barbaresi, Katusic, Colligan, Weaver, & Jacobsen, 2007; The MTA Cooperative Group, 1999). The two other long-term risks investigated by scientific studies, drug abuse and antisocial behaviours, were much less mentioned by the French press beside 2005. Indeed, on 22 September 2005, the French National Institute of Medical Research (INSERM) released a report on antisocial behaviours in children and adolescents. This report asserted that ADHD children are at an increased risk of antisocial behaviour when they reached adolescence. It recommended the screening of all French three-year-old children to identify those at risk of ADHD and to treat them to prevent future antisocial behaviour. The first line treatment recommended in this report combines behavioural therapies with various social measures, but medication was also considered. This report was hotly debated in the French press, especially by psychoanalysts. For them, children expressing their suffering with symptoms of conduct disorders are in need of care, but they disagree with the systematic screening of all three-year-old children. According to them, the predictive value of such a screening is low and would expose many young children to unjustified medication and stigma.

**Table IV.** Long-term risks associated with ADHD according to the French press.

	1995–2000	2001–2005	2006–2010	2011–2015	Total
Antisocial behaviour	2	7	1	2	12
Drug abuse	0	6	3	4	13
School failure	7	19	15	18	59

### Does medication protect ADHD children from academic underachievement?

While French press articles frequently mentioned school failure as a major concern for ADHD children (59 articles), only 11 of these articles explicitly suggested that medication can protect children from this risk (Table V). In addition two articles gave voice to experts defending conflicting opinions about this protective effect (Table V). In agreement with the scientific consensus (Sharpe, 2014), only four articles stated that medication does not protect from the long-term risk of academic underachievement. All four articles cited US scientific studies that supported this negative conclusion. For example *L’Express* (29 March 2004) reported: “Studies about the long-term outcomes of methylphenidate treatment show no benefit regarding academic underachievement and social integration compared to untreated ADHD sufferers.” In contrast, among the 11 articles suggesting that medication is protective, six reported only on individual cases of ADHD children that improved their school performances after starting medication. A typical example from *La Croix* (7 May 2013) illustrates this rhetoric: “Julien, who for long was not able to benefit from a standard school, is now medicated. With the drug this 10-years old boy is able to concentrate, to keep up at school and regains his self-confidence.” The five remaining articles put this type of assertion: “Medication can prevent school exclusion and the fall of school performances” (*Le Monde*, 19 June 2013).

**Table V.** Does medication protect ADHD children from school failure?

	1995–2000	2001–2005	2006–2010	2011–2015	Total
Yes	1	3	2	5	11
No	1	2	0	1	4
Yes + no	0	2	0	0	2

### Is brain imaging able to reveal ADHD?

Only one article (*Le Progrès*, 26 November 2002) suggested that brain imaging is able to detect differences between ADHD children and controls. According to this article, brain metabolism is increased in the frontal region of ADHD children. It is likely that this article echoed a study published in November 1990 in *The New England Journal of Medicine* claiming that glucose metabolism is depressed in the frontal region of ADHD adults. Indeed, this study has been widely covered by US newspapers, while the subsequent studies that disconfirmed this initial claim did not attract newspapers’ attention (Gonon et al., 2012). In contrast, two articles published in *Libération* said that brain imaging does not reveal obvious differences that characterize ADHD (*Libération*, 31 August 2009 and 12 February 2015). The remaining 156 articles we examined did not mention brain imaging.

### ADHD representation depending on the political orientation of French newspapers

According to reports from the French Institute of Public Opinion (IFOP) the nine journals we examined were distributed in three groups according to their main political orientation: *Le Figaro, L’Express* and *Le Point* are right wing, *Le Progrès, Le Sud Ouest* and *La Croix* are at the centre and *Le Monde, Libération* and *Le Nouvel Obs* are left wing. We tested whether this political orientation is associated with differences in the number of articles supporting each ADHD model (Table VI). We observed that the six newspapers with a right or centre orientation tend to support more frequently the biomedical model than the three newspapers at the left wing (2 × 3 chi^2^ test *p* = 0.034). However, the percentage of articles exclusively supporting the psychodynamic understanding of ADHD is low and does not depend on the political orientation (Table VI). In this table the number of articles is given in parentheses as a percentage of the whole number of articles within each political orientation.

**Table VI.** ADHD representation in function of the political orientation.

	Right wing	Centre	Left wing	Total
Biomedical	37 (74%)	39 (70.9%)	28 (51.9%)	104
Psychodynamic	8 (16%)	8 (14.5%)	10 (18.5%)	26
Both views	5 (10%)	8 (14.5%)	16 (29.6%)	29
Total	50	55	54	159

### The role of the drug industry

Social studies about ADHD often express the opinion that the pharmaceutical industry strongly contributes to creating and extending the market for ADHD drugs (Lloyd & Norris, 1999; Phillips, 2006). However, the evidence that this industry directly influences the media is scarce. Lloyd and Norris (1999) cited a few newspaper articles mentioning drug companies and ADHD drugs, but these articles actually reported financial news about these companies. They were not directed at the general public and did not deal with ADHD prevalence, diagnosis or therapeutic options.

Among the French newspaper articles the influence of the pharmaceutical companies on the prescription of psychostimulant drugs was mentioned 15 times, but only in the context of the USA. These articles warned that the US drug industry contributed to the rapid increase in ADHD medication in several ways, including their financial support to pro-drug associations of parents. In France there is only one association of parents of ADHD children (Association TDAH-France). Its president since 2002 is very active. She has been cited by 15 articles of our corpus and defends a nuanced view of the biomedical model that acknowledges the complexity of ADHD etiology (Edwards, Howlett, Akrich, & Rabeharisoa, 2014). The website of this association mentions that it received grants from SHIRE, a drug company that sells an extended-release form of methylphenidate in France. However, none of the articles we examined mentioned this financial support.

Only one epidemiological study investigated the ADHD prevalence in France. This study, published in 2011 by French and US scientists, concluded that ADHD occurs in 3.5–5.6% of French youth (Lecendreux et al., 2011). The authors emphasized that this range is similar to that of other countries. Since 2011, these prevalence data have been cited by 21 newspaper articles. However, none of them mentioned that they resulted from a telephone survey supported by a grant from SHIRE Development Corporation, although this support was acknowledged in the scientific publication (Lecendreux et al., 2011).

## Discussion

### Comments about ADHD representation in French newspapers

We have classified newspaper articles into three categories: those mainly defending the biomedical model of ADHD, those mainly supporting the psychodynamic understanding and those that gave voice to both views. We observed that about two-thirds mainly supported the biomedical model. It must be noted, however, that this model is a default option of our coding: articles that did not included traits characterizing the psychodynamic model were classified in this first category. This explains why 24 out of the 104 articles classified as supporting the biomedical model nevertheless expressed fear and criticisms about psychostimulant medication. If we exclude these articles of the first category, 59% (80/135) of French newspaper articles actually supported the biomedical model.

In line with this, several experts defending the biomedical model nevertheless emphasized that ADHD is a real disorder but not a disease. Neurological dysfunctions are put forward in only 26 articles and genetic factors are said to play a major role in only eight articles. Taking into account that five articles highlighted both alleged biological causes, only 29 articles out of the 133 articles classified as defending either the biomedical model alone, or both models, put forward biological arguments in support of the biomedical model. Moreover, these alleged neurogenetic arguments were less frequent in the recent period (2011–2015). Thus, according to most French newspapers, ADHD is a real syndrome that may require medication in combination with other therapeutic approaches, but not a neurological disease that can be treated by a drug.

Studies investigating ADHD representation in US and Swedish newspapers during the 1990s concluded that the general press favours the biomedical model (Börjesson, 1999; Schmitz et al., 2003). In contrast, Horton-Salway (2011) showed that, during 2000–2009, the psychosocial understanding of ADHD was dominant in the UK newspapers. Our study of French newspapers during these two decades might explain this discrepancy. We observed that during the most recent period the proponents of the biomedical model became more prudent and refrained from putting forward weak biological arguments. As a result, the biomedical model defended by the vast majority of French newspaper articles became more nuanced.

Finally, according to French newspapers, the psychosocial model of ADHD appears to put emphasis on the persons (i.e. children and parents) rather than on the society and this might reflect the influence of psychoanalysis in France. In line with our observation, Horton-Salway (2011, p. 533), examining the UK newspapers, also concluded that both models “perform a common function in representing families as in need of regulation”.

### Comparison of the general and the specialized press in France

Considered as a whole, the discourses about ADHD in the French general press and in the specialized press directed at social workers were similar in several aspects. First, a small minority of articles put forward neurological and/or genetic arguments in support of the biomedical model. Second, a few articles asserted that medication protects ADHD children from school underachievement. Third, both discourses remained almost silent about the role of major social factors such as poverty, school policies and the influence of the pharmaceutical industry. However, the psychodynamic understanding of ADHD dominated in the specialized press (56%) whereas it did not in the general press (35%) (Ponnou et al., 2015). Moreover, articles of the specialized press either defended the biomedical model or the psychodynamic one but debates were rare. In contrast, dialogues and debates between experts defending either model took place in the general press (18%).

### Comparison between newspaper coverage and television

In contrast, the discourse about ADHD in French TV programmes widely differed from that of the general and specialized press. First, 16 out of 60 French TV programmes discussed the contribution of genetic factors to ADHD and 11 of them claimed that ADHD is a genetic disease without giving voice to opposite opinions (Bourdaa et al., 2015). Second, six TV programmes showed brain scans on screen and erroneously claimed that brain imaging can reveal ADHD. In contrast, only one article of the general press and none of the specialized press said so. Third, 22 TV programmes discussed the effectiveness of medication as protecting from school failure and 16 of them erroneously claimed that it is very effective without giving voice to the opposite opinion. In contrast, only 11 out of 159 newspaper articles and two out of 93 articles of the specialized press defended, without debate, this erroneous opinion. Fourth, unsurprisingly, TV programmes did not mention the deleterious effect of excessive TV exposure. Finally, our TV study did not explicitly quantify whether the psychodynamic understanding of ADHD was represented. However, we can say that among the 41 distinct experts that were invited to give their opinion in TV programmes, psychoanalysts were less numerous than in the general press. All four TV experts that were often invited by TV producers (three to six programmes) defended the biomedical model (Bourdaa et al., 2015). These experts were also the authors of articles in the specialized press and were interviewed by journalists of the general press. We noted that they were much more prudent in the press than in their TV appearance (Ponnou et al., 2015). However, we must acknowledge that our comparison between press and TV suffers from a limitation: we did not investigate on TV programmes broadcast since January 2011. We do not know whether ADHD representation has evolved in the more recent TV programmes.

### Limitation: the Internet as a source of information concerning ADHD

One major limitation of our studies is that we did not investigate the role of the Internet, although it does play a role, at least through the website maintained by the TDAH-France association (Edwards et al., 2014). In 1998 the first source of information about ADHD among US parents was the doctor followed by newspapers and teachers (Bussing, Schoenberg, & Perwien, 1998). In 2008 the four main sources were the Internet, health professionals, newspapers, and television (Bussing et al., 2012). Moreover, in 2008, the Internet was by far the most preferred source of information (Bussing et al., 2012). However, this study did not specify whether the Internet was an initial source or the preferred source for those seeking additional information. Several experts mentioned in French newspaper articles that requests for consulting skyrocketed just after every broadcast of a TV programme about ADHD and this suggests that television might provide initial information that may possibly trigger additional Internet searches. However, this hypothesis needs further investigation.

## Conclusion

The biomedical model of ADHD exclusively focuses on the biology of the patient and promotes medication as the most appropriate treatment. French TV programmes mainly defended this strict biomedical model. In contrast, the French press, either general or specialized, mainly defended a more complex understanding of ADHD etiology and treatment. Even medical experts supportive of ADHD medication mainly defended in the press, but not in TV programmes, a nuanced opinion about ADHD: the familial and social contexts must be taken into account, medication can help but in combination with educative and therapeutic approaches. Unfortunately, surveys show that TV programmes represent the main source of information about health for Europeans (European Commission, 2007). It remains to elucidate why French TV programmes differ from newspapers articles and if the same difference occurs in other countries.

One might also wonder what medical doctors who defended a nuanced view of the biomedical model in newspapers, but a strictly biological one on TV, actually say to parents. In France the prescription of methylphenidate is constrained: the first prescription, and its renewal each year, must be done by a psychiatrist in a public hospital. We have heard that prescription practices widely vary between hospitals. This may suggest that the discourse to parents that is put forward to justify medication, or its absence, also widely varies and this might deserve investigation.

## Supplementary Material

Ponnou-data.xlsx
